# Paramedian Forehead Flap Raised in the Base of Previously Used Median Forehead Flap Pedicle

**Published:** 2016-09

**Authors:** Mohammadreza Akhoondinasab, Roohollah Sobhani

**Affiliations:** Department of Plastic Surgery, Hazrat Fatemeh Hospital, Iran University of Medical Sciences, Tehran, Iran

**Keywords:** Paramedian forehead flap, Carcinoma, Basal cell, Axial artery

**DEAR EDITOR**

Nose is the most common site of facial skin cancer because of its continuous sun exposure.^1^ Soft tissue tumors such as basal cell carcinoma (BCC) are the most common tumors of this region. The choice of treatment for BCC is surgical demolitions. Since these tumors grow more aggressively and are so likely to recur from the involved surgical margins, the surgery requires often wide resection of the midfacial region’s structures to obliterate adequate margin.^2^ Thus reconstruction surgery of defected regions plays an important role in the integrity of complex facial expressions, facial functions and the aesthetic outcome. In the present study, we described an eccentric case of a recurrence of a BCC managed by a paramedian forehead flap in an unusual condition.

## CASE REPORT

**Fig. 1:**
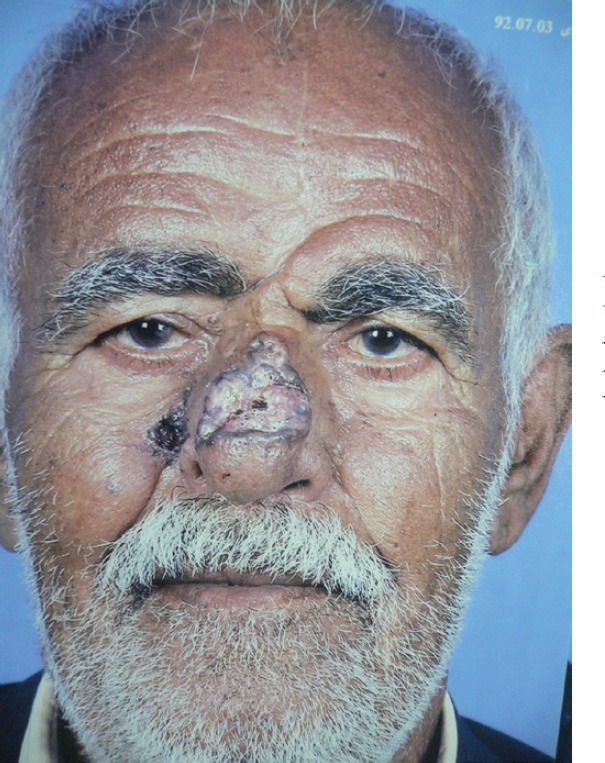
Recurrence of a BCC on previously reconstructed nasal defect using median forehead flap

The patient is an 85-year old man, who was referred to our center for BCC recurrence after previous resection in almost 30 years ago (Figure 1) . He underwent an incisional biopsy of lesion, with the result of BCC. In the mentioned surgery, median flap was used to cover the defected area. After a careful clinical and radiological evaluation, a surgical treatment with the demolition of the lesion and a wide removal of the region was planned in order to attain a safe margin. In the latter resection, at first, mucosal and osteocartilaginous lining were made and then paramedian flap in a size of 9×8 cm, raised from the left side of forehead, was used for coverage. Each of the supratrochlear arteries was absent because they have been sacrificed in the previous median flap surgery.

As it is well known, vascular pedicles in both sides of nasofrontal zone, containing supratrochlear arteries as axial, is the basis of median flap. If the stalk of flap is dissected for any reasons, other vessels and their multiple anastomoses supply forehead and supraorbital zones. In this case, we used paramedian flap in base of previous median flap pedicle. Since all of the cardinal arteries were dissected in the previous surgery, as the narrowed remained pedicle, the blood supply of the used paramedian forehead flap was only based on blood supply from a rich plexus of the anastomosing vessels from the terminal branches of angular artery in the nasal bridge. 

**Fig. 2:**
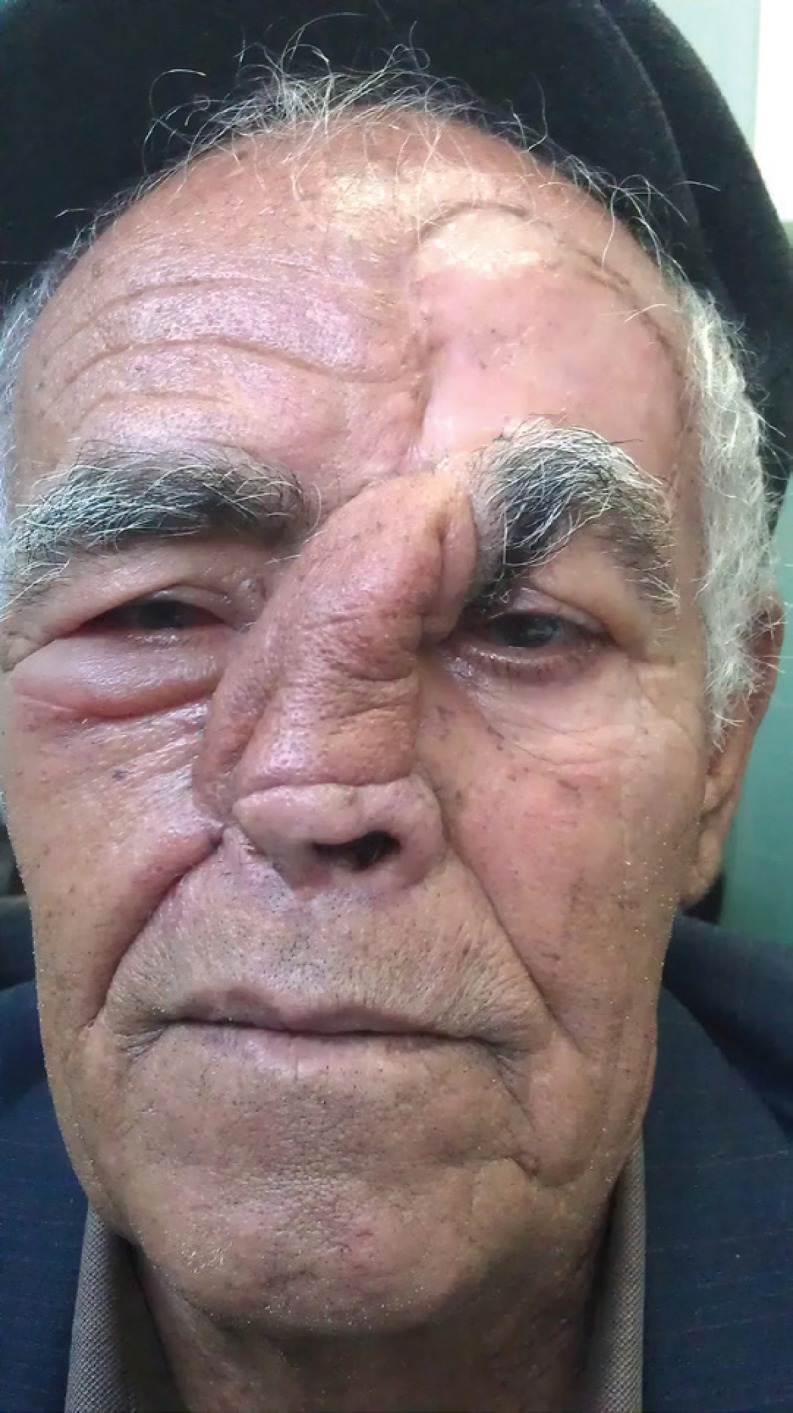
No evidence of abnormalities or recurrence of the tumor after long term follow-up.

Unlike the absence of a certain arterial supply in the flap pedicle there was not any ischemic event after the flap raised and inset. Then the patient candidate for three stage flap operation. The long term follow-up did not show evidence of any abnormalities of the wound or recurrence of the tumor (Figure 2). Median and paramedian forehead flaps have been recommended for insetting of defects greater than 2.5-3 cm in diameter, especially when the cartilage framework has been ravaged; for their success, it is important to preserve the vascular pedicle of these flaps (supratrochlear artery) and obtain sufficient thinning of the subcutaneous tissue from the distal flap. The paramedian forehead flap is a useful flap with a resilient vascular supply that is largely used for reconstruction of complex or large nasal defects.^3^^-^^5^

In conclusion, for special occasions, to inset in defected area, it is acceptable to raise paramedian flap in base of previously used flap pedicles, without the presence of any axial arteries and only in base of rich anastomotic arterial plexus in the nasofrontal angle of each side. 

## CONFLICT OF INTEREST

The authors declare no conflict of interest.
